# Impaired brain function improved by l-carnitine in patients with cirrhosis: evaluation using near-infrared spectroscopy

**DOI:** 10.1038/s41598-020-70585-y

**Published:** 2020-08-11

**Authors:** Hiroyuki Nakanishi, Yuka Hayakawa, Youhei Kubota, Masayuki Kurosaki, Leona Osawa, Kento Inada, Sakura Kirino, Koji Yamashita, Shuhei Sekiguchi, Mao Okada, Wang Wan, Mayu Higuchi, Kenta Takaura, Chiaki Maeyashiki, Shun Kaneko, Nobuharu Tamaki, Yutaka Yasui, Takamasa Noda, Kaoru Nakanishi, Kaoru Tsuchiya, Jun Itakura, Yuka Takahashi, Namiki Izumi

**Affiliations:** 1 Department of Gastroenterology and Hepatology, Musashino Red Cross Hospital, 1-26-1 Kyonan-cho, Musashino, Tokyo 180-8610 Japan; 2 Department of Psychiatry, National Center Hospital, National Center of Neurology and Psychiatry, Tokyo, 187-8551 Japan; 3 Department of Psychiatry, Japan Depression Center, Rokubancho Mental Clinic, K-PLAZA 2F, 1-7, Rokubancho, Chiyoda-ku, Tokyo, 102-0085 Japan

**Keywords:** Liver cirrhosis, Neuro-vascular interactions, Encephalopathy

## Abstract

To evaluate the effects of l-carnitine on impaired brain function in patients with liver cirrhosis. We conducted a retrospective cohort study that included sequential 80 liver cirrhosis patients with impaired brain function evaluated using near-infrared spectroscopy (NIRS). Among them, l-carnitine was administered to 48 patients. The NIRS data and blood ammonia level at baseline and after 8 weeks of treatment were compared between patients administered with l-carnitine (l-carnitine group) and those who were not (control group). The NIRS data at baseline were similar between the l-carnitine and control groups (0.04 ± 0.04 vs. 0.04 ± 0.05 mMmm, p = n.s), whereas those in the l-carnitine group (n = 48) were significantly better than that of the control group at 8 weeks of treatment (n = 32) (0.103 ± 0.081 vs. 0.040 ± 0.048 mMmm, p < 0.001). In the l-carnitine group, 35.4% (17/48) of patients had hyperammonemia. The NIRS data of the l-carnitine group at 8 weeks of treatment were significantly improved than that of the control group, irrespective of baseline ammonia levels (0.11 ± 0.09 vs. 0.04 ± 0.05 mMmm, p = 0.005, and 0.10 ± 0.06 vs. 0.02 ± 0.03 mMmm, p = 0.003, for normal baseline ammonia and elevated ammonia levels, respectively). In the multivariate analysis, l-carnitine administration (odds ratio [OR] 3.51, 95% confidence interval [CI] 1.23–9.99, p = 0.019) and baseline NIRS data of ≤ 0.07 mMmm (OR 5.21, 95% CI 1.69–16.0, p = 0.0041) were found as independent significant factors. l-carnitine improves impaired brain function in patients with liver cirrhosis.

## Introduction

Hepatic encephalopathy is defined as a brain dysfunction caused by liver insufficiency or portosystemic shunting^1^ and is characterized by a broad spectrum of neurological or psychiatric abnormalities, ranging from subclinical alterations to coma^1^. Overt hepatic encephalopathy was recognized by the detection of marker symptoms, such as disorientation and flapping tremor, because of their excellent interobserver reliability^2,3^. The positron emission tomography (PET) was used to examine brain oxygen consumption and cerebral blood flow and revealed that patients with overt hepatic encephalopathy and those with liver cirrhosis without overt hepatic encephalopathy had a lower cerebral metabolic rate of oxygen and cerebral blood flow than healthy subjects^4^. Impaired cerebral blood flow and cerebral oxyhemoglobin metabolism in hepatic encephalopathy could be improved^5^. Since the cerebral ammonia uptake remains the same between the state of hepatic encephalopathy and recovery^5^, not only the blood ammonia level but also the brain function should be evaluated in patients with hepatic encephalopathy.

Covert hepatic encephalopathy consists of minimal and grade 1 hepatic encephalopathy^1^, could impair the cognitive function, lower work efficiency, reduce the quality of life^6,7^, impair driving skills^8–11^, or results in poor prognosis^12–14^. Some cases of covert hepatic encephalopathy were difficult to evaluate and diagnose, because of lacking reliable diagnostic clinical signs. Although several screening tools and neuropsychological tests were developed^2,12,15–31^, brain functional imaging methods for patients with covert hepatic encephalopathy were not established in daily clinical settings. Therefore, a readily available and reliable methodology of functional brain imaging is urgently required in not only patients with overt but also with covert hepatic encephalopathy.

Recently, near-infrared spectroscopy (NIRS) was developed as a readily available brain functional imaging technology in daily clinical settings^32–38^. Due to the advancements of neurological studies, brain function can be evaluated by monitoring dynamic changes of cerebral blood flow according to neurovascular coupling theory, i.e., neurons, astrocyte, and arterial vessel communication^39–41^. NIRS could noninvasively measure the regional cerebral oxygenated hemoglobin (oxyhemoglobin), deoxyhemoglobin, and total hemoglobin concentration. The time resolution of NIRS is higher than that of PET and functional magnetic resonance imaging (MRI). NIRS is also portable, does not have any restrictions in posture, and is flexible in setting tasks. Therefore, brain function can be evaluated based on dynamic changes in regional cerebral oxyhemoglobin concentration in response to a given task. This is essential to assess a latent brain function abnormality. NIRS could also evaluate the regional cerebral oxyhemoglobin metabolism and cerebral blood flow by combining with MRI^34^.

Some reports showed the usefulness of NIRS in patients with liver disease^37,38^. Especially, patients with minimal hepatic encephalopathy showed a lower increase of cerebral oxyhemoglobin concentration in response to word fluency task than that of patients with liver cirrhosis without minimal hepatic encephalopathy^37^. In patients with hepatic encephalopathy, the impaired reaction of cerebral oxyhemoglobin concentration might occur due to astrocyte swelling and dysfunction^42–44^ because some reports showed that astrocyte controls the regional cerebral blood flow and provides energy sources such as ketone body and carnitine to the neuron^39,41^. l-Carnitine is also reported to improve hyperammonemia and hepatic encephalopathy^45,46^, however, whether l-carnitine can improve those impaired neurons, astrocyte, and vascular interaction remains unknown.

Liver cirrhosis patients were generally considered as having secondary l-carnitine insufficiency. Some patients received l-carnitine replacement therapy by the attending physician's discretion. In all the patients with cirrhosis, l-carnitine therapy is covered by a national insurance system in Japan. Although the definition of carnitine insufficiency in liver cirrhosis patients remains unclear, the symptoms of carnitine deficiency are thought to be muscle cramps, general fatigue, hepatic encephalopathy, and hyperammonemia in liver cirrhosis patients. Therefore the liver cirrhosis patients who suffered from these symptoms were considered to have a possibility of carnitine deficiency. Some attending physicians advised those patients who had impaired brain function to receive l-carnitine supplementation, but some patients did not want to receive it mainly because of avoiding polypharmacy and the cost of medicine.

Therefore, this study aimed to elucidate the effects of l-carnitine on impaired brain function in patients with liver cirrhosis using NIRS.

## Results

**Figure 1:**
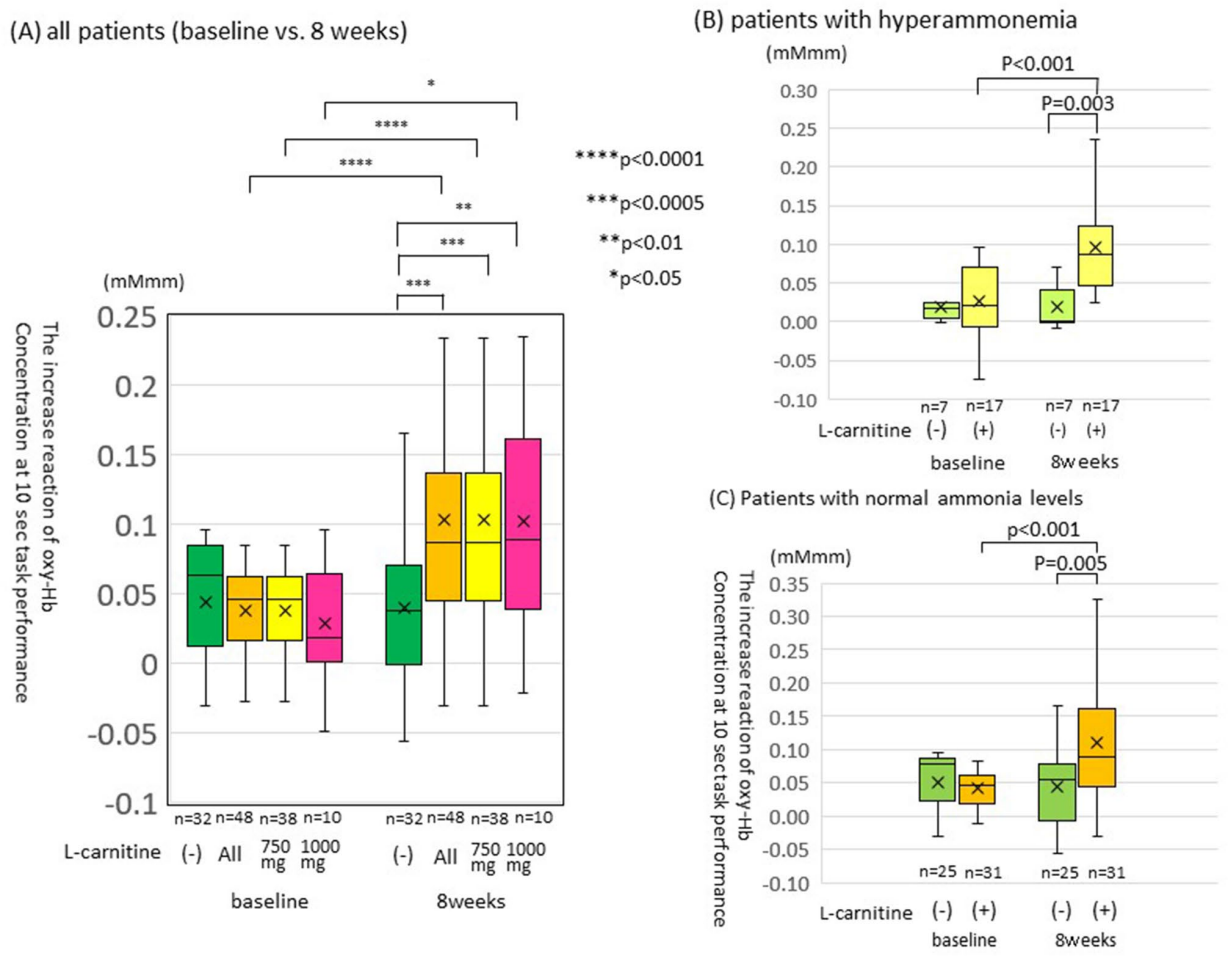
Comparison of NIRS data between the l-carnitine and no treatment groups at baseline and 8 weeks post-treatment. (**A**) All patients. The box plot includes the stratified analysis according to the l-carnitine dose. (**B**) Patients with hyperammonemia. (**C**) Patients with normal ammonia levels at baseline.

At baseline, no significant difference in age, gender, etiology, branched-chain amino acid administration, and serum total carnitine level between the two groups (Table 1). However, the albumin level was lower, the bilirubin level was higher, and Child–Pugh grade was more aggravated in the l-carnitine than that in the control group. Although baseline NIRS data were not different between the two groups, they were significantly better in the l-carnitine group (n = 48) than that in the control group at 8 weeks post-treatment (n = 32) (0.103 ± 0.081 vs. 0.040 ± 0.048 mMmm, p < 0.0005, Fig. 1A). The NIRS data at 8 weeks treatment were significantly improved as compared to baseline in the all patients of l-carnitine group (n = 48) (0.103 ± 0.082 vs. 0.036 ± 0.035 mMmm, t(47) = − 5.47, p < 0.0001, Fig. 1A), but not in the control group (n = 32) (0.040 ± 0.048 vs. 0.044 ± 0.053 mMmm, t(31) = 0.37, p = 0.72). In the stratified analysis, the same results were obtained between patients receiving 750 mg a day of l-carnitine and those of 1500 mg a day (0.104 ± 0.084 vs. 0.038 ± 0.034 mMmm, t(37) = − 4.73, p < 0.0001, and 0.102 ± 0.079 vs. 0.029 ± 0.044 mMmm, t(9) = − 2.65, p = 0.026, Fig. 1A).

**Table 1:** Patient characteristics.

	l-Carnitine group n = 48	Control group n = 32	p value
Age	71.2 ± 9.6	72.7 ± 8.6	0.43
Male gender [n (%)]	18 (42.9)	15 (60.0)	0.21
**Etiology (%)**			
ALD	12 (25.0)	6 (18.8)	0.85
HBV	3 (6.2)	1 (3.1)	
HCV	24 (50.0)	18 (56.2)	
NBNC	9 (18.8)	7 (21.9)	
**L-Carnitine dose (%)**			
750 mg	38 (79.2)	0	
1500 mg	10 (20.8)	0	
BCAA at baseline [n (%)]	24 (50.0)	9 (28.1)	0.065
Viable HCC [n (%)]	7 (14.6)	6 (18.8)	0.76
**Child–pugh class (%)**			
A	34 (70.8)	28 (87.5)	0.024
B	14 (30.4)	3 (9.4)	
C	0 (0.0)	1 (3.1)	
Albumin (g/dL)	3.50 ± 0.52	3.77 ± 0.59	0.034
Total bilirubin (mg/dL)	1.25 ± 0.87	0.80 ± 0.30	0.006
NH_3_ (μg/dL)	54.5 ± 31.9	48.1 ± 29.4	0.38
Platelet count (× 10^3^/μL)	120 ± 105	114 ± 54	0.77
Prothrombin time (%)	82.3 ± 17.9	89.9 ± 16.3	0.066
Total carnitine (μmol/L)	60.8 ± 16.7	64.1 ± 11.4	0.37
Free carnitine (μmol/L)	47.6 ± 13.1	51.0 ± 8.9	0.25
Acylcarnitine (μmol/L)	13.1 ± 5.9	13.1 ± 5.0	0.98
NIRS data (mMmm)	0.036 ± 0.035	0.044 ± 0.053	0.56
NCT-A (sec)	46.4 ± 23.5	46.1 ± 11.8	0.95
NCT-B (sec)	97.0 ± 47.2	99.1 ± 41.7	0.85
SMI (cm^2^/m^2^)	43.9 ± 6.2	42.6 ± 6.1	0.44

*HBV* hepatitis B virus, *HCV* hepatitis C virus, *ALD* alcoholic liver disease, *BCAA* branched-chain amino acid, *NIRS* near-infrared spectroscopy, *NCT* number connection test, *SMI* skeletal muscle mass index.

**Figure 2:**
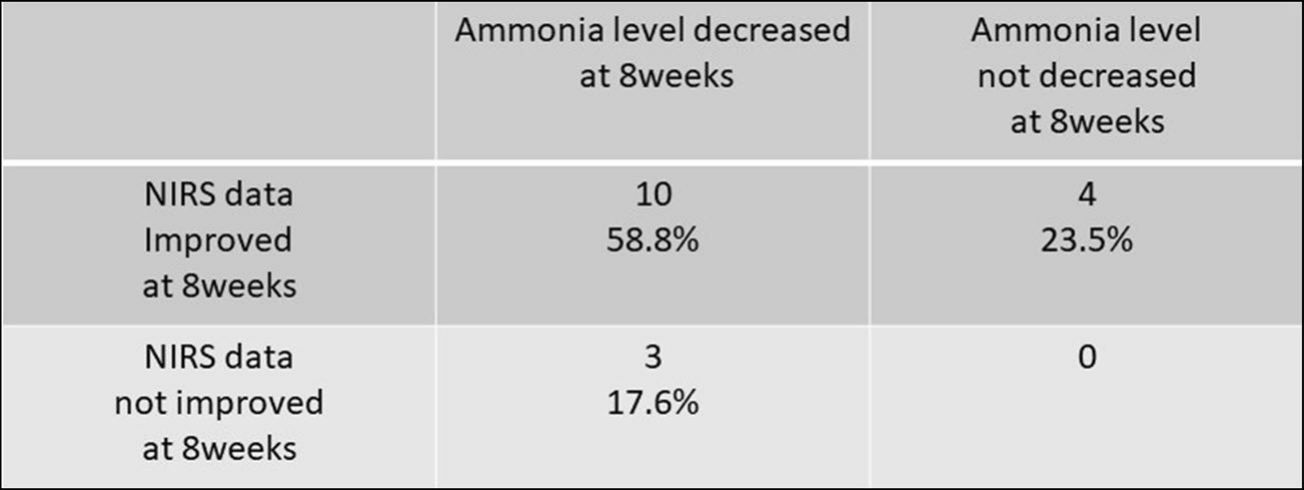
The treatment effect of l-carnitine on patients with hyperammonemia. Effects of l-carnitine on hyperammonemia and impaired brain function in patients with liver cirrhosis with hyperammonemia were shown in this chart.

In the l-carnitine group, 35.4% (17/48) of patients had hyperammonemia at baseline. Among them, 82.4% (14/17) showed NIRS data improvement, and 76.5% (13/17) showed decreased serum ammonia levels at 8 weeks of treatment. In 58.8% (10/17) of patients, NIRS data were improved and ammonia levels were decreased, whereas 23.5% (4/17) of patients showed improved NIRS data without decreasing serum ammonia levels at 8 weeks. No patients had unimproved NIRS data or NH_3_ levels (Fig. 2).

In patients with hyperammonemia at baseline (n = 24), the NIRS data in the l-carnitine group (n = 17) was significantly better than that in the control group (n = 7) (0.10 ± 0.06 vs. 0.02 ± 0.03 mMmm, p = 0.003, Fig. 1B). In the l-carnitine group, the NIRS data at 8 weeks treatment was significantly improved as compared to the baseline (0.096 ± 0.058 vs. 0.027 ± 0.050 mMmm, p < 0.001, Fig. 1B).

**Figure 3:**
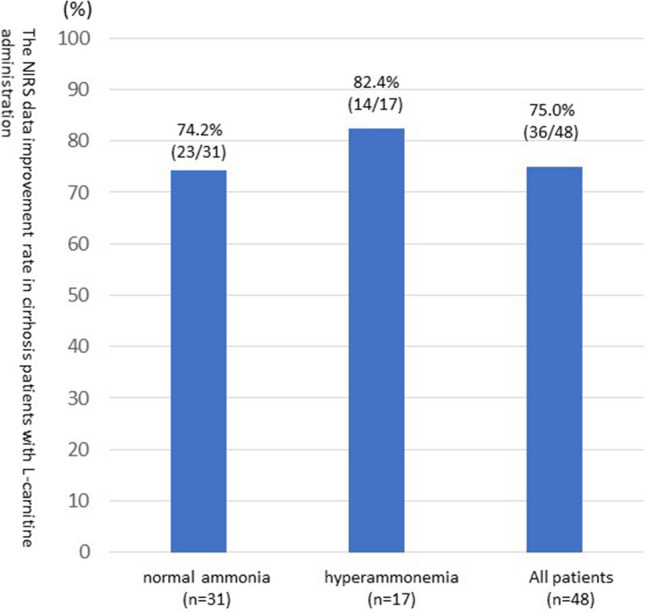
NIRS data improvement rate according to the baseline ammonia levels in patients with cirrhosis administered with l-carnitine.

In the l-carnitine group, 74.2% (23/31) of patients with impaired brain function and with normal ammonia levels had improved NIRS data at 8 weeks of treatment (Fig. 3).

In patients with baseline normal ammonia levels, the NIRS data of l-carnitine group (n = 31) at 8 weeks of treatment were significantly better than that of the control group (n = 25) (0.11 ± 0.09 vs. 0.04 ± 0.05 mMmm, p = 0.005, Fig. 1C). In the l-carnitine group (n = 31), the NIRS data at 8 weeks of treatment were significantly improved as compared to the baseline (0.110 ± 0.093 vs. 0.042 ± 0.025 mMmm, p < 0.001, Fig. 1C).

In the univariate analysis, factors associated with improved NIRS data were l-carnitine administration (p = 0.0038) and baseline NIRS data (p = 0.0010) (Table 2). In the multivariate analysis, l-carnitine administration (OR 3.51, 95% CI 1.23–9.99, p = 0.019) and baseline NIRS data of ≤ 0.07 mMmm (OR 5.21, 95% CI 1.69–16.0, p = 0.0041) were found as significant independent factors (Table 3).

**Table 2:** Univariate analysis of factors associated with NIRS data improvements.

	Univariate		
	Odds ratio	95% CI	p
Age > 70 y.o	2.55	0.88–7.35	0.083
Female gender	1.25	0.51–3.06	0.26
**Etiology (%)**			
HBV	1.00	0.39–2.52	1.00
HCV	2.23	0.72–6.92	1.00
Others	1.43	0.38–5.44	0.60
Baseline BCAA	1.15	0.46–2.86	0.77
l-Carnitine	4.07	1.57–10.50	0.0038
Child–Pugh B or C	1.62	0.55–4.79	0.38
Albumin ≤ 3.5 g/dL	1.58	0.64–3.34	0.32
Total bilirubin > 0.9 mg/dL	1.43	0.57–3.60	0.45
Ammonia > 50 μg/dL	1.80	0.69–4.69	0.23
Platelet count < 100 × 10^3^/μL	1.42	0.57–3.52	0.45
Prothrombin time < 80%	1.08	0.40–2.92	0.89
Total carnitine < 68 μmol/L	1.63	0.53–5.04	0.40
Free carnitine > 52 μmol/L	2.10	0.71–6.21	0.18
Acylcarnitine < 15.5 μmol/L	1.59	0.53–4.80	0.41
NIRS data ≤ 0.07 mMmm	6.53	2.26–19.01	0.0010
NCT-A < 42 s	1.45	0.57–3.66	0.43
NCT-B < 98 s	2.09	0.80–5.46	0.13
Skeletal muscle mass depletion	0.65	0.19–2.1	0.48

**Table 3:** Multivariate analysis of factors associated with NIRS data improvements.

	Multivariate		
	OR	95% CI	p
l-Carnitine	3.51	1.23–9.99	0.019
Baseline NIRS data < 0.07 mMmm	5.21	1.69–16.0	0.0041

No patient had adverse effects of l-carnitine during the study period.

## Discussion

Brain function is recently thought to be an interaction among neurons, astrocytes, and cerebral arterial vessels^39,40^. Astrocyte especially controls the local cerebral blood flow to supply neurons with oxygen and essential nutrients depending on the neural activity^39,41^. The pathophysiology of hepatic encephalopathy is associated with impaired interaction between astrocytes, neurons, cerebral blood flow, and cerebral oxygen consumption^4,5,43,47–49^. This neurovascular coupling could be elucidated by monitoring the dynamic changes of the regional cerebral arterial blood flow. Based on this perspective, brain functional imaging modalities such as PET, SPECT, functional MRI, and NIRS are thought to be useful^50^. NIRS is a portable, easy to set, and has a high time resolution of 0.1 s and is recently reported to be a useful tool to evaluate patients with impaired brain function with liver cirrhosis^37^. In the present study, NIRS was used to evaluate the effects of l-carnitine on brain activity, which improved after 8 weeks of l-carnitine treatment in patients with cirrhosis.

l-Carnitine transports long-chain free fatty acids into the mitochondria, subsequently producing ATP by β-oxidation and TCA cycle and metabolizing ammonia by activating the urea cycle via *N*-acetylglutamate. Acyl CoA transforms into acetyl CoA through β-oxidation. l-carnitine synthesizes acetylcarnitine from acetyl CoA. Further, acetylcarnitine facilitates acetylcholine production in the striatum and hippocampus^51^.

Therrien et al. reported that l-carnitine reduces the concentration of ammonia and lactate in the cerebrospinal fluid in a study of portocaval-shunted rats^52^. The brain avoids ammonia toxicity by converting α-ketoglutarate into glutamate and glutamine synthesis. In another clinical study, l-carnitine treatment for 6 months reduced blood ammonia levels and improved NCT-B scores in patients with liver cirrhosis with covert hepatic encephalopathy^53^. Another study showed that 45.8% (11/24) of patients with liver cirrhosis with minimal hepatic encephalopathy were ameliorated by l-carnitine administration for 3 months^54^.

In a randomized, double-blind placebo-controlled trial, the 90-day l-carnitine treatment (2,000 mg twice a day) improved hyperammonemia and hepatic encephalopathy^45^. A retrospective analysis of 34 patients with overt hepatic encephalopathy found that oral administration of l-carnitine reduced overt hepatic encephalopathy recurrence^55^. These data suggest that l-carnitine may improve overt or covert hepatic encephalopathy.

In the present study, l-carnitine improved the NIRS data and reduced blood ammonia concentration after 8 weeks of treatment. In the l-carnitine group, 58% of patients with impaired brain function with hyperammonemia had ameliorated NIRS data. Moreover, 23.5% of patients in the l-carnitine group had improved NIRS data without decreased blood ammonia concentration. Conversely, in patients with impaired brain function with normal ammonia levels, NIRS data were significantly improved in the l-carnitine group than that in the control group. Therefore, l-carnitine might improve the brain function not only by improving hyperammonemia but also by other mechanisms.

Recently Wang et al.^56^ reported that intracellular reactive oxygen species and all amino acids, including glutamine, were increased in the NH_4_Cl-treated human astrocyte; however, these reactions were reduced with l-carnitine treatment. Moreover, in the NH_4_Cl-treated human astrocytes, increased concentration of 3-methyl-2-oxovaleric acid, which was ammonia induced and played a crucial role in neurological impairment, was significantly decreased with l-carnitine co-treatment. Accordingly, l-carnitine might improve hepatic encephalopathy by not only lowering ammonia concentration but also by directly affecting the brain.

There are some limitations in this study. There is a risk of bias due to non-randomized controlled design and retrospective analysis. But we started observation of patients from the date of NIRS. Therefore we retrospectively analyzed the prospective observational data. And patients were sequentially enrolled. The randomized controlled trial is needed.

Although there was not a statistically significant difference, there were more patients who had BCAA supplementation in the l-carnitine group (50%) compared to the control group (28%). However, there was no patient who started BCAA supplementation during the study period. BCAA has been shown to positively affect sarcopenia, which is an independent risk factor for hepatic encephalopathy itself^57^. In the present study, baseline SMI between the two groups was not different, and baseline skeletal muscle mass depletion was not associated with NIRS data improvement at 8 weeks in this cohort. Further studies are needed in this issue.

In conclusion, l-carnitine administration improves impaired brain function in liver cirrhosis patients.

## Methods

The brain function of 140 patients with liver cirrhosis was evaluated using NIRS between June 2013 and October 2019. The diagnostic value of impaired brain function was set as an increased reaction of oxyhemoglobin concentration increased to word fluency task at 10 s, i.e., ≤ 0.1 mMmm using NIRS according to a recent report that compared NIRS and EEG abnormality in liver cirrhosis patients^37^. Among them, 80 patients showed impaired brain function. No patients had a history of mental disorders including alcohol abuse and intake of antidepressants or other psychotropic drugs. All patients underwent brain computed tomography or MRI and had no apparent brain structural diseases, such as brain infarction, brain tumor, etc.

All patients performed the number connection test A and B using neuropsychological tests^21^. We analyzed baseline skeletal muscle mass index (SMI) with the CT method using the Slice-O-Matic version 5.0 Software program (Tomovision, Montreal, Canada). Skeletal muscle area was measured on the axial image at the level of the third lumbar vertebra (L3). Skeletal Mass Index (SMI) was calculated by dividing the muscle area (cm^2^) with a square of height (m^2^)^58^. The definition of skeletal muscle mass depletion was based on the guideline described by the Japan Society of Hepatology (42 cm^2^/m^2^ in men and 38 cm^2^/m^2^ in women).

**Figure 4:**
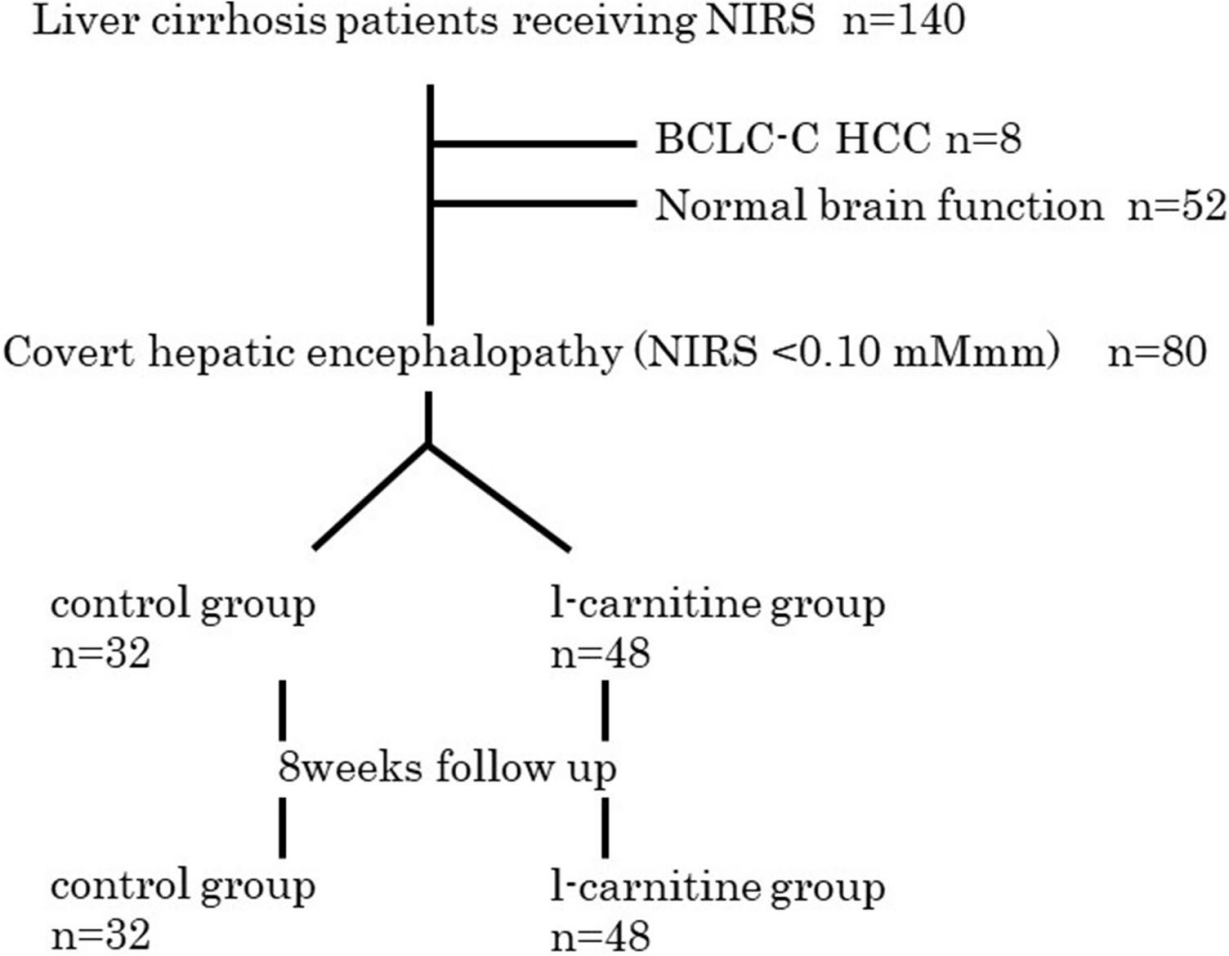
Patients’ flow chart.

A total of 48 patients were administered 250 mg or 500 mg of l-carnitine three times a day for 8 weeks (l-carnitine group), according to the attending physician’s discretion, and the remaining 32 patients were not (control group) (Fig. 4). The study was performed in accordance with the Helsinki Declaration and approved by the ethics committee of Musashino Red Cross Hospital. Informed consent was obtained from all participants.

### Assessment of brain activity

**Figure 5:**
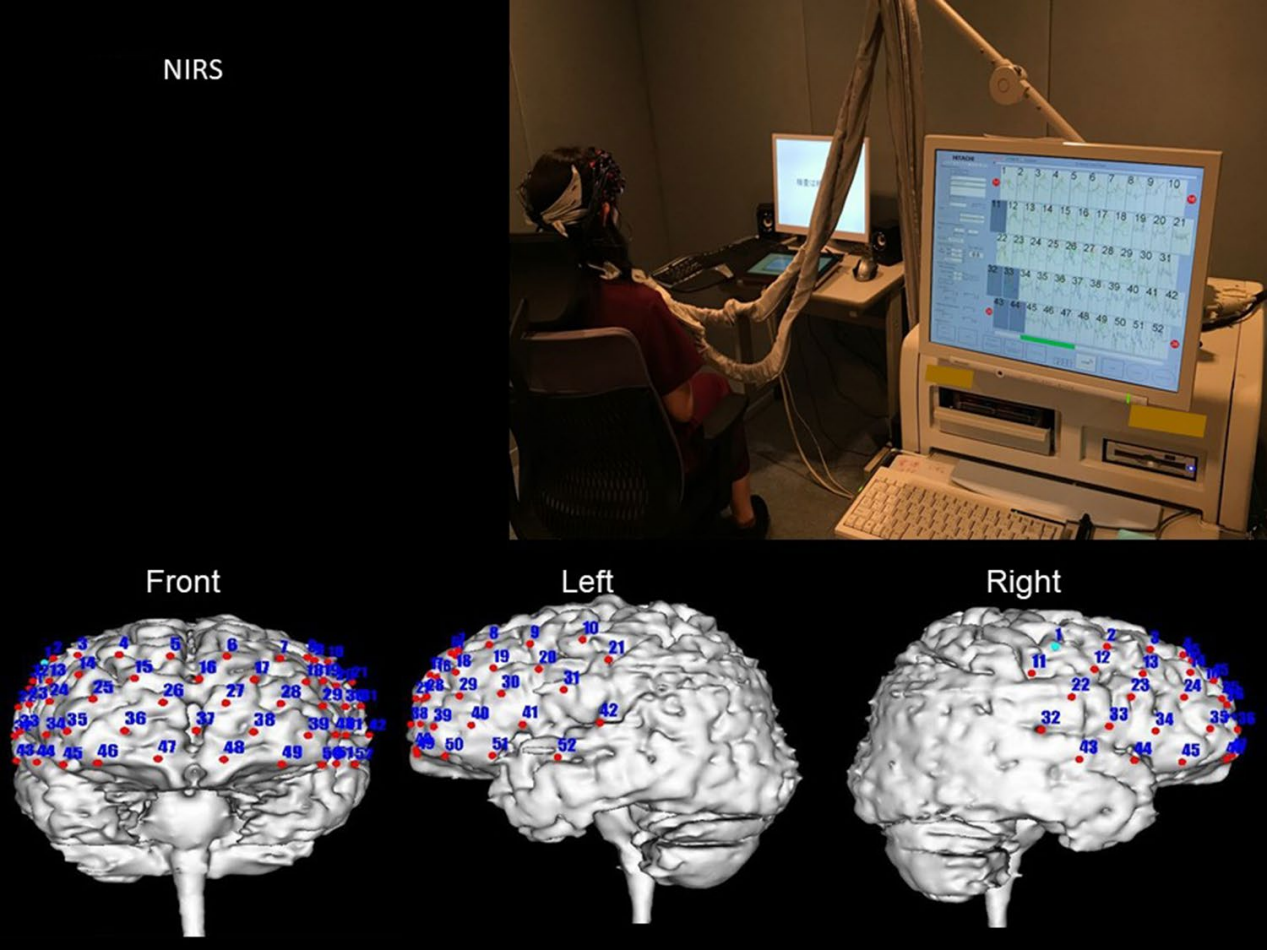
Near-infrared spectroscopy.

The brain function was evaluated using NIRS. The regional cerebral oxyhemoglobin concentration was measured using a 52-channel NIRS machine (Hitachi ETG4000, Hitachi Medical Cooperation, Tokyo, Japan, Fig. 5). NIRS captures changes in regional cerebral oxyhemoglobin concentration every 0.1 s. For each of the 52 channels, optic fiber devices are connected to an application probe placed on the patient’s scalp. The 52 channels cover the frontal, upper temporal, and anterior parietal lobes of the brain. The reflected light is detected by a probe positioned 30 mm away from the application probe. Changes in oxyhemoglobin concentration could be calculated by measuring the reflected light^32^. In this study, data were measured using 7 channels, which were previously reported to diagnose mental disorders and minimal hepatic encephalopathy (channels 36–38 and 46–49), were selected for the analysis^35,37,59,60^. The mean increase in oxyhemoglobin concentration of these 7 channels at 10 s during word fluency task was measured. Moreover, changes from baseline to 8 weeks were compared between the two groups.

### Statistical analysis

Statistical analyses of the clinical data were performed using Fisher’s exact probability test, Student’s *t* test, and a paired *t* test. We conducted univariate and multivariate logistic regression analysis to obtain the factors associated with improved NIRS data. A p-value of < 0.05 was considered statistically significant. All statistical analyses were performed using EZR software version 2.30 (Saitama Medical Center, Jichi Medical University, Saitama, Japan).

## Data Availability

Requests for data and materials should be addressed to the corresponding author.
